# The Gut–Immune–Brain Axis: An Important Route for Neuropsychiatric Morbidity in Inflammatory Bowel Disease

**DOI:** 10.3390/ijms231911111

**Published:** 2022-09-21

**Authors:** Rebecca Katharina Masanetz, Jürgen Winkler, Beate Winner, Claudia Günther, Patrick Süß

**Affiliations:** 1 Department of Molecular Neurology, University Hospital Erlangen, Friedrich-Alexander-Universität Erlangen-Nürnberg (FAU), 91054 Erlangen, Germany; 2 Department of Stem Cell Biology, University Hospital Erlangen, Friedrich-Alexander-Universität Erlangen-Nürnberg (FAU), 91054 Erlangen, Germany; 3 Center of Rare Diseases Erlangen (ZSEER), University Hospital Erlangen, Friedrich-Alexander-Universität Erlangen-Nürnberg (FAU), 91054 Erlangen, Germany; 4 Deutsches Zentrum Immuntherapie (DZI), Friedrich-Alexander-Universität Erlangen-Nürnberg (FAU), 91054 Erlangen, Germany; 5 Department of Internal Medicine 1, University Hospital Erlangen, Friedrich-Alexander-Universität Erlangen-Nürnberg (FAU), 91054 Erlangen, Germany; 6 Department of Neurology, University Hospital Erlangen, Friedrich-Alexander-Universität Erlangen-Nürnberg (FAU), 91054 Erlangen, Germany

**Keywords:** inflammatory bowel disease, systemic inflammation, gut-brain axis, gut microbiota, neuroinflammation, depression, Crohn’s disease, ulcerative colitis

## Abstract

Inflammatory bowel disease (IBD) comprises Crohn’s disease (CD) and ulcerative colitis (UC) and is associated with neuropsychiatric symptoms like anxiety and depression. Both conditions strongly worsen IBD disease burden. In the present review, we summarize the current understanding of the pathogenesis of depression and anxiety in IBD. We present a stepwise cascade along a gut–immune–brain axis initiated by evasion of chronic intestinal inflammation to pass the epithelial and vascular barrier in the gut and cause systemic inflammation. We then summarize different anatomical transmission routes of gut-derived peripheral inflammation into the central nervous system (CNS) and highlight the current knowledge on neuroinflammatory changes in the CNS of preclinical IBD mouse models with a focus on microglia, the brain-resident macrophages. Subsequently, we discuss how neuroinflammation in IBD can alter neuronal circuitry to trigger symptoms like depression and anxiety. Finally, the role of intestinal microbiota in the gut–immune–brain axis in IBD will be reviewed. A more comprehensive understanding of the interaction between the gastrointestinal tract, the immune system and the CNS accounting for the similarities and differences between UC and CD will pave the path for improved prediction and treatment of neuropsychiatric comorbidities in IBD and other inflammatory diseases.

## 1. Introduction

Inflammatory bowel disease (IBD) is a chronic inflammatory disease comprising two major clinical entities—Crohn’s disease (CD) and ulcerative colitis (UC). IBD incidence remains constantly high in industrialized countries and continuously rises in emerging economies. The burden of IBD to societies in Europe and North America is growing despite its constant incidence, as increased life expectancy leads to a higher number of years IBD patients live with disability [1,2,3]. 

A major contribution of IBD-associated disease burden is conferred by the high prevalence of neuropsychiatric comorbidity. Recent clinical data indicate prevalence of anxiety and depression in IBD patients of 32.1% and 25.2%, respectively [4]. Anxiety and depressive symptoms are more prevalent in CD than in UC, and both patients with active disease and female patients are more frequently affected [4,5]. Vice versa, patients suffering from depression are more commonly diagnosed with IBD, and anxiety and depressive symptoms are significantly associated with adverse outcomes related to disease activity in IBD [5]. In addition, genetic predisposition for depression correlates with the development of IBD [6]. These epidemiological observations strongly suggest a bidirectional link between IBD and neuropsychiatric disease burden. Therefore, understanding the causative mechanisms leading to CNS involvement in IBD and tailored treatment strategies might ameliorate both neuropsychiatric and gastrointestinal symptoms, thereby significantly improving patients’ quality of life.

Recent data imply that the CNS manifestation in IBD is mediated by a systemic immune reaction, which is propagated to the brain. In line with this, biologicals targeting inflammatory circuits exerted robust positive effects on depressive symptoms in many autoimmune diseases [7,8], and in IBD in particular [9]. Therefore, research in recent years increasingly focused on the characterization of local and systemic immune reactions in IBD, and entry routes of inflammation into the CNS as well as their influences on brain-resident immune and glia cells. The ultimate aim is to understand how these changes impair the function of neurons to cause neuropsychiatric symptoms.

In the present review, we propose a gut–immune–brain axis as the underlying anatomical and functional route leading to depression and other neuropsychiatric symptoms in IBD. We will review the current understanding of IBD pathophysiology and the development of systemic inflammation in IBD. Next, we will shed light on different anatomical routes conveying this inflammation to the CNS. We propose that these routes do not represent passive barriers, but are dynamically regulated and actively contribute to CNS immune activation. We will then focus on the current knowledge and open questions regarding neuroinflammation in distinct brain regions of preclinical IBD models. In addition, we will highlight the present understanding of neuronal pathology and behavioral impairment in IBD, and how these are linked to CNS immune activation. Eventually, we will shed light on how the gut microbiota may interfere with different stages of the gut–immune–brain axis to promote neuropsychiatric morbidity in IBD.

## 2. Etiology and Pathophysiological Hallmarks of IBD

IBD is a multifaceted disease with a dissimilar clinical presentation and pathology of CD and UC. Both entities share common features including rectal bleeding, diarrhea, epithelial erosion, and distorted crypt architecture, but exhibit distinct inflammatory patterns and differ in their spatial manifestations [10,11]. Discontinuous transmural inflammation, predominantly affecting the terminal ileum, caecum, colon, and perianal area is characteristic for CD, whereas continuous mucosal inflammation and ulceration constrained to the colon and rectum are present in UC.

The etiology of IBD is multifactorial and heterogeneous, influenced by the interaction between environmental, genetic, and microbial cues. Several internal and external environmental factors such as psychosocial stress, diet, pollutants, and toxins have been associated with IBD onset and progression [12]. Exposure of the intestine to diverse endogenous or exogenous factors causes aberrant immune responses mediating inflammation and inducing intestinal damage. Disruption and destabilization of the intestinal epithelial barrier function is a key ‘catalysator’ to IBD pathogenesis, enabling the influx of intraluminal microbes, metabolites, and antigens into the subepithelial layers of the gut, thereby promoting local inflammation [13,14]. In addition to intestinal epithelial barrier defects, dysfunction of the gut vascular barrier was observed in IBD, causing increased infiltration of blood-derived inflammatory cells [14].

From an immunological perspective, IBD is characterized by mucosal immune dysregulation including traits of loss of immune tolerance associated with uncontrolled immune responses towards antigens derived from the commensal intestinal microbiota [14]. Large genome-wide association studies (GWAS) identified more than 240 susceptibility loci, many of which contain genes encoding for proteins inducing adaptive and effector immune functions [15]. IBD susceptibility loci contain genes in immune signaling pathways including tumor necrosis factor (TNF), interleukin (IL)-12, IL-23, and integrins, all important targets for interventional approaches in IBD [15]. Immunological hallmarks of CD include activation of antigen presenting cells (APCs) inducing the differentiation of naïve CD4+ T helper (Th) cells into effector cell subsets Th1, Th17, and regulatory T cells (Tregs). In contrast, APCs in UC drive naïve CD4+ T cell differentiation into Th2, Th9, and Treg effector subsets [10].

Another pivotal contributor to IBD pathophysiology is the enteric nervous system (ENS). Particularly, enteric glial cells (EGCs), a heterogeneous population of peripheral neuroglia within the ENS, regulate intestinal immunity, tissue integrity, and barrier function in mouse models of intestinal inflammation [16]. Recent studies have demonstrated massive remodeling in the ENS compartment during intestinal inflammation in IBD patients [17]. Future studies will further elucidate the tight interplay of the ENS with the gut microbiota and immune processes in IBD.

Collectively, IBD is a chronic autoimmune disease caused by the interaction of different etiological factors and characterized by dysregulated immune processes and barrier function in the gastrointestinal tract.

## 3. Animal Models for Studying IBD In Vivo

Several preclinical models mimicking important pathophysiological hallmarks of IBD are used to study the human disease. Most commonly, large intestinal inflammation (colitis) is induced by treatment with chemicals like dextran sulfate sodium (DSS), 2,4,6-trinitrobenzene sulfonic acid (TNBS), and oxazolone (OXA) [18]. DSS-induced colitis is subdivided into an acute colitis induced by short-term application of DSS via the drinking water for several days, and chronic colitis induced by repetitive DSS treatment cycles interrupted by remission phases with administration of normal drinking water [17,19]. Besides chemically induced models of IBD, further strategies include adoptive cell transfer, microbial infection, and genetic modification [20]. Of note, the development, progression, severity, spatial distribution, and pathophysiology of gastrointestinal inflammation, as well as associated extraintestinal comorbidities, vary among the different models, partly explaining conflicting results reported.

The majority of IBD studies in rodents are conducted using models with chemically induced intestinal inflammation. These models are easy to induce, reproducible and expeditiously exhibit symptoms resembling human IBD [18]. Most commonly, experimental colitis is chemically induced by oral DSS administration or intrarectal TNBS instillation in mice. However, there is great variation in treatment paradigms for acute and chronic colitis induction of chemically treated experimental models. Further, colitogenicity and elicited immunological responses vary among species, strains, and gender and are strongly dependent on environmental factors [21,22]. The severity in DSS-induced colitis depends not only on the dosage, but also the molecular weight of the substance [23], thereby impeding comparability of results from different studies. Both DSS and TNBS are lethal at high dosages, have toxic effects on intestinal epithelial cells (IECs) by inducing cell death and intestinal damage, and disrupt the integrity of the mucosal barrier [19]. DSS induces barrier dysfunction, translocation of bacterial antigens and provokes infiltration of particularly innate immune cells into the lamina propria [23,24]. Overall, clinical manifestation of DSS-induced colitis resembles human UC. In contrast, TNBS induces excessive immune responses and inflammation predominated by Th1 and Th17 cells, representing some clinical features of human CD [18]. Noteworthy, none of the present animal models used can mimic the full clinical and pathological spectrum of human IBD, limiting their translational potential. Few genetic models have been established that more closely model CD pathology. The *Tnf^ΔARE^* model harbors a deletion encompassing the Tnf AU-rich element (ARE), resulting in unrestrained Tnf expression that provokes intestinal inflammation reminiscent of human CD [25]. In the last decade, novel and more sophisticated mouse models were generated by specifically deleting proteins in IECs, such as *Casp8^ΔIEC^* [26], *Setdb1^ΔIEC^* [27], and *Fadd^ΔIEC^* [28]. These distinct genetic deletions promote programmed necrosis of epithelial cells leading to inflammatory reactions, primarily innate immune responses, in the gastrointestinal tract comprising inflammatory cytokine expression and infiltration of myeloid cells into the lamina propria [14,27,28]. Interestingly, these models feature CD-like ileitis including Paneth cell dysfunction, origin of inflammation in the ileum and environmental-dependent disease manifestation. However, they are not as extensively characterized and as easily available as chemically induced models.

Collectively, the heterogeneity of human IBD is reflected by a growing number of rodent models for experimental colitis, all of which represent different aspects and subforms of the complex human disease.

## 4. Gut Microbiota in IBD

Data derived from both human IBD patients and preclinical IBD models indicate a pivotal role of the gut microbiota in IBD development and progression.

Dysbiosis of many bacterial taxa has long been observed in stool and gut tissue samples of IBD patients [29,30]. The pathophysiological relevance of the gut microbiota in IBD is being increasingly acknowledged. Mechanistically, the gut microbiota interact with the intestinal epithelium, but also induce T cell responses in the gut and microbiota-specific T cells of the thymus as shown in mice [31,32]. Disturbance of these processes under IBD-related dysbiosis may result in impaired barrier functions and increased autoimmune reactivity by altered tolerance induction [32]. Moreover, altered production of microbiota-derived metabolites during IBD fuels local immune responses. For instance, short chain fatty acids (SCFAs) are a group of small-molecule lipid mediators secreted by bacteria, including acetate, propionate, and butyrate. They regulate the development of B cells and the differentiation of Treg cells and are involved in inflammasome activation [33]. The fecal content of SCFAs is markedly decreased in IBD patients [34]. Besides SCFAs, microbial dysbiosis impairs bile acid and tryptophan metabolism, which can further augment intestinal inflammation [33]. Taking these findings together, bacterial dysbiosis and imbalance of bacterial metabolites represent major factors driving IBD but offer the opportunity to define novel therapeutic targets [35].

While most studies focused on the implication of bacteria, less abundant commensals have also been found to play a considerable role in IBD. These include bacteriophages [36,37], archaea [38], and eukaryotes like fungi, helminths and protozoa [39,40,41]. For example, a common intestinal eukaryote, *Blastocystis*, impacts gut bacterial composition and immune responses, and long-term colonization has been suggested to be protective against intestinal inflammation [40]. Interestingly, CD and UC show disease-specific enrichment or deprivation of certain fungal genera [41]. In DSS- and *Citrobacter rodentium*-induced colitis, mucosal, but not luminal fungi conferred intestinal barrier protection by inducing IL-22 in Th17 cells [42]. Moreover, distinct *Candida albicans* species were enriched in the intestinal mucosa of UC patients and exacerbated colitis via induction of IL-1β and secretion of candidalysin, which triggers a Th17 response [43].

Overall, reduced diversity as well as altered composition of microbiota create a distinct microbial fingerprint characteristic for CD and UC patients [36,44]. More importantly, microbiota and mycobiota regulate intestinal immunity and barrier function directly or via distinct metabolites, thereby contributing to IBD pathophysiology [42,43,44].

## 5. Systemic Inflammation in IBD

Beyond the gastrointestinal tract, mounting evidence indicates a circulatory inflammatory signature in IBD patients and its respective preclinical models [14,45,46].

Systemic inflammation is considered a major prerequisite for the observed CNS comorbidity in IBD patients. It can be elicited in a three-step sequence: (I) First, chronic inflammatory processes in the gut lead to a dysfunctional intestinal epithelial barrier. Under homeostasis, this barrier is upheld by intercellular tight junctions and covered by a thick mucus layer [47]. In line with this, most barrier-forming tight junction proteins are downregulated in experimental colitis in rodents as well as in IBD patients [46,48]. In addition, dysbiosis induces impairment of the mucus barrier, accompanied by increased epithelium damage, which further promotes bacterial translocation and inflammation [47,49,50]. The resulting intestinal hyperpermeability enables microbial infiltration into the lamina propria. (II) Secondly, local immune cells in the lamina propria recognize microbial structures by pattern recognition receptors (PRRs), leading to local inflammatory activation characterized by the production of signaling molecules (cytokines) and chemoattractants (chemokines) [14,44]. Moreover, immune cells are primed in the Peyer’s patches, specialized lymphatic structures of the lamina propria and submucosa, to exert immune functions at distant sites. These cells express gut-homing receptors, ensuring return to the gut for later adaptation. (III) In a third step, gut-primed immune cells, inflammatory molecules, gut microbes and their metabolites enter the bloodstream. This requires a leakage of the local gut vascular barrier (GVB) [51,52]. The GVB consists of endothelial cells connected by intercellular tight junctions and adherens junctions, and ensheathed by EGCs and pericytes. The barrier function of the GVB is dependent on the canonical wingless-type, catenin-beta 1 (Wnt/β-catenin) signaling pathway and prevents passive diffusion of molecules larger than 4 kDa [52]. In IBD, GVB leakage is facilitated by the microenvironmental inflammatory milieu, involving interferon (Ifn)-γ [46,51]. Increased permeability of the gut vascular barrier also enables evasion of circulatory inflammatory cells to the gut, thereby initiating a self-perpetuating mechanism.

The stepwise transition from gastrointestinal to systemic inflammation is evidenced by bacterial translocation and increased levels of circulating inflammatory mediators, both in IBD patients and respective in vivo models.

In the DSS-induced colitis model, inflammatory markers (S100A8/A9) and cytokines (e.g., Il-1β, Il-6, Il-17a) are elevated in the serum compared to controls (Table 1, [19,53,54,55,56]). Of note, serum inflammatory markers exhibit intraday volatility and gender-specific differences [19,55]. Compared to acute DSS-induced colitis, chronic DSS paradigms lead to a switch of serum immunophenotype towards a more Th2-like pattern with increased levels of Il-4 and Il-10, while levels of Ifn-γ and Il-6 remain elevated [19]. Additionally, serum cytokine profiles differ between DSS- and TNBS-induced colitis. The latter display lower levels of Il-6, but higher levels of Il-12p40, Il-23p19, and Il-17 [19].

Interestingly, serum levels of Il-1β, Tnf, and Il-6 in DSS-induced colitis are further increased by transient hypoxia [54]. Mechanistically, hypoxia inducible factor-1α (HIF-1α), a transcription factor mediating the hypoxic response in cells and tissue, is a potential upstream regulator of gastrointestinal and systemic inflammation [54]. HIF-1α activation and expression is increased in IBD patients and animal models and suppressing HIF-1α activation through vitamin D receptor signaling ameliorated TNBS- and DSS-induced colitis [57]. Moreover, gut barrier disintegration, bacterial dissemination from the gut into lymphoid organs (mesenteric lymph nodes, spleen), and systemic Tnf levels in DSS-induced colitis was reduced by treatment with the HIF-1α inhibitor CG-598 [58]. Thus, HIF-1α may be a promising therapeutic target to reduce local and systemic inflammation in IBD.

**Table 1:** Major studies on IBD-related neuroinflammation and associated systemic immune changes, neuronal alterations, and behavioral phenotypes.

Reference	Genotype [Initial Age]	Colitis Induction	Systemic Inflammation	Neuroinflammation	Neuronal Changes	Behavior
**DSS-induced colitis**						
[59]	C57BL/6 mice [8w]	2.5% DSS for 5d + 6d H_2_O (acute)	−	↔ Il-1β, Il-6	−	−
		2.5% DSS on d1-5, d12-16, d23-27 + 3d H_2_O (chronic)	↑ Hmgb1	↑ Il-1β, Il-6, Hmgb1 (Hc), Il-10 (Hc, Cb) ↑ Gfap^+^ astrocytes, reactive morphology (Hc) ↑ Caspase-1, Gsdm (Cb, Hc), caspase-11 (Hc)	↓ Brain activity ↓ Manganese uptake (Hc)	↓ Long-term memory
[60]	Male C57BL/6 mice [10-11w]	2.5% DSS for 4d/7d (acute)	↑ Tnf-α, Il-1β, Il-6, Il-10	↑ Mϕs, monocytes (Hc) ↑ Gfap^+^ astrocytes (Hc) ↑ CD45^low^CD11b^+^Iba1^+^ microglia (Hc) ↑ Tnf, Il-1β, Il-6, Il-10 (Hc)	↑ Neurogenesis (Hc): ↑ Proliferation (Ki67) ↑ Maturation (Dcx) Altered NSC cell cycle	−
		2% DSS on d1-7, d21-28, d42-49 (chronic)	↑ Il-6, Il-10	↑ Mϕs, monocytes (Hc) ↑ Iba1^+^ myeloid cells (Hc) ↑ Tnf, Il-1β, Il-6, Il-10 (Hc)	↓ Neurogenesis (Hc): ↓ Cell migration, integration	↓ Exploratory behavior ↓ Spatial learning
[53]	C57BL/6 mice [10-12w]	3% DSS for 1d and 3d + 2d H_2_O (acute)	↑ Monocytes (d1, d3) ↑ Il-6 (d5)	↑ Mϕs (d3, d5), monocytes (d5) ↑ Tmem119^+^ microglia (d3) ↑ Tmem119^+^ MHC-II^+^CD86^+^ microglia (d5) ↑ Iba1^+^ cell amoeboid morphology (Hc)	−	−
		1.8% DSS for 7d + 14d H_2_O (acute + remission)	−	↑ Iba1^+^ cell amoeboid morphology (acute, Hc)	−	↓ Exploratory behavior ↓ Mobility (acute)
[56]	C57BL/6 mice [16w]	2% DSS for 5d + 2d H_2_O (acute)	↑ Il-6, Cxcl1, S100A8/A9	↑ Caspase-1 ↑ *Tnf*, *Il-1*β, *Cxcl1*, *Lcn-2*, *S100A8/A9* ↑ Neutrophils, monocytes ↑ Iba1-IR	↓ *Bdnf*	−
[61]	C57BL/6J mice [6-8w]	3% DSS for 6d (acute)	↑ Tnf, Ccl2, Il-6, Ifn-γ	↑ *Tnf*, *Il-1*β, *Il-6*, *Vcam-1* ↑ Monocytes, neutrophils, Mϕs ↔ CD45^lo^CD11b^+^Cx3cr1^hi^ microglia, CD68-IR ↓ MHC-II^+^ microglia	−	↑ Flurothyl-induced seizure susceptibility (reversed by anti-neutrophil and anti-Tnf treatment)
		3% DSS for 6d + 30d H_2_O (acute + remission)	−	↑ Monocytes, neutrophils	−	↑ Flurothyl-induced seizure susceptibility
		3% DSS on d1-6, d22-27, d44-49 + 7d H_2_O (chronic)	−	↑ Monocytes, neutrophils, Mϕs	−	
[54]	Male C57BL/6 mice [7-8w]	3% DSS for 5d (acute)	↑ Il-6	↑ Iba1-IR (Hc) ↑ *Il-1*β, *Il-6*	−	−
		3% DSS for 5d (acute) + hypoxia (6000 m) for 2d	↑ Il-1β, Il-6	↑ Iba1-IR (Cx, Hc) ↑ *Tnf*, *Il-1*β, *Il-6*	−	−
[62]	C57BL/6 mice [3w]	2% DSS for 5d + 5-7w H_2_O (acute + remission)	−	↑ *Iba1*, *Il-1*β, *iNos2*, *Nod1*, *Nod2*, *Tlr2*, *Tlr4*, *Il-17ra* (Hc) ↑ Iba1^+^ cells, amoeboid morphology (Hc)	↓ Neurogenesis (Hc): ↔ Proliferation (Ki67) ↓ Maturation (Dcx)	↓ Object recognition ↑ Anxiety
[63]	Male C57BL/6N mice [n.a.]	2% DSS for 7d (acute)	−	↓ Iba1-IR (Cx, Hc, MeA, Pvh), CD68-IR (Hc, MeA) ↓ *Nos2*, *Iba-1*, *CD11b*, *CD206* (Cx) ↑ *Ido-1*, *Tnf*, *CD86*, *Chil3* (Cx) ↑ CD45^hi^CD11b^+^ myeloid cells	−	−
[64]	C57BL/6J mice [64w]	1% DSS on d1-5, d8-12, d15-19, d22-26 + 11d H_2_O (chronic)	−	↑ Nlrp3 activation (brain, Men) ↑ Iba1^+^ myeloid cells (Cx, Hc) ↑ Gfap^+^/C3^+^ astrocytes (Cx, Hc) ↑ gut-derived T cell accumulation (Men)	↓ Map2-IR (Cx, Hc) ↓ NeuN^+^ neurons (Cx, Hc)	↑ Anxiety ↓ Spatial memory ↓ Object recognition
[24]	C57BL/6 mice [7-8w]	5% DSS for 7d (acute)	↑ Il-6	↑ Iba1-IR (Cx) ↑ *Il-6*, *Tnf* (Cx)	−	−
[65]	Female C57BL/6 mice [8w]	3% DSS on d1-5 + 2d H_2_O (acute)	↑ Tnf, Il-6	↑ Iba1-IR, Il-6-IR (Hc) ↑ *Tnf*, *Il-1*β (Hc)	−	−
		3% DSS on d1-5, d8-12, d15-19, d22-26 + 3d H_2_O (chronic)	↑ Il-6	↑ Gfap-IR (Hc) ↑ *Tnf*, *Il-1*β, *Gfap* (Hc)	↓ Neurogenesis (Hc): ↓ Proliferation (Ki67) ↓ Maturation (Dcx)	−
[66]	Male C57BL/6 mice [24-40w]	2% DSS on d1-3, d18-20 + 20d H_2_O (chronic)	−	↓ Gfap-IR (Hc) ↑ Iba1-IR, ↔ CD68-IR (Hc) ↔ Il-6-IR, ↔ Cox-2-IR	−	↔ Locomotor activity ↔ Anxiety
[67]	Male ddY mice [6-7w]	1.5% DSS for 7d (acute)	−	↑ Iba1^+^ cells, amoeboid morphology (Hc) ↑ Gfap^+^ astrocytes, reactive morphology, IR (Hc)	↓ NeuN-IR (Hc) ↓ Neurogenesis (Hc): ↓ Proliferation (BrdU) ↓ Maturation (Dcx)	↑ Depressive-like behavior
[68]	Male Wistar rats [n.a.]	5% DSS for 6-8d (acute)	−	↑ *Nos2* (Cx, Hc, Ht), *Il-6* (Cx) ↑ iNOS-IR, 3-NT-IR (Me) ↑ Iba1^+^ cells, amoeboid morphology, IR (Me)	↑ Ventricular volume ↑ FosB/∆FosB-IR (Nac, Drn)	↓ Exploratory behavior ↑ Anhedonia
		5% DSS for 6-8d + 2-3d/7-10d H_2_O (acute + remission)			↑ Ventricular volume ↑ FosB/∆FosB-IR (Drn)	↑ Anhedonia ↑ Anxiety ↑ Depressive-like behavior
**Non-DSS-induced colitis**						
[69]	Male NMRI mice [n.a.]	6 mg DNBS *i.r.* + 3d recovery	−	↑ *Tlr2*, *Tlr4*, *Myd-88*, *Hmgb1*, *Tnf*, *Il-6* (Hc) ↑ ROS production, ↓ GSH, ATP (Hc)	↓ *Bdnf* (Hc)	↑ Anxiety ↑ Depressive-like behavior
[70]	Male NMRI mice [n.a.]	10 mg TNBS *i.r.* + 3d recovery	−	↑ *Nos2*, *Tnf* (Hc) ↑ nitrite	−	↑ Depressive-like behavior (reversed by NO inhibition)
[71]	Male Sprague-Dawley rats [n.a.]	25 mg TNBS *i.l.* + 4d or 10d recovery	−	↑ Iba1^+^ cell amoeboid morphology (d4, Erc, Hc) ↔ Iba1^+^ cell amoeboid morphology (d10, Erc, Hc) ↑ Tnf (Hc)	−	↑ Pentylenetetrazole-induced seizure susceptibility
[61]	C57BL/6J mice [6-8w]	T cell transfer-colitis: Splenic naïve CD4^+^ T cells (5*10^5^) + Tregs (1*10^5^)	↑ Tnf, Ccl2, Il-6, Ifn-γ	↑ Monocytes, T cells	−	↑ Flurothyl-induced seizure susceptibility

Studies only focusing on behavioral changes or on the expression of individual inflammatory genes in the brain were excluded. Most studies were performed using mice with DSS-induced colitis and differ in the applied experimental paradigm, partly explaining spatiotemporally diverging findings in the CNS. DSS-treatment paradigms were divided into acute (short-term DSS administration ≤ 8d) and chronic (long-term DSS administration in cycles) colitis. The brain region showing a particular immune response is indicated in brackets. If not indicated, changes were measured in the whole brain, and gender of experimental mice was not specified in the publications. ATP: adenosine triphosphate; Bdnf: brain-derived neurotrophic factor; C3: complement factor 3; Cx: Cortex; d: days; Dcx: doublecortin; DNBS: dinitrobenzene sulfonic acid; Drn: dorsal raphe neucleus; DSS: dextran sulfate sodium; Erc: entorhinal cortex; Gsdm: gasdermin; GSH: glutathione; Hc: hippocampus; Hmgb1: high-mobility group box 1; Ht: Hypothalamus; Il: interleukin; i.r.: intrarectal; IR: immunoreactivity; Mϕ: macrophage; Me: median eminence; MeA: medial amygdala; Men: meninges; n.a.: not available; NAc: Nucleus accumbens; NMRI: Naval Medical Research Institute; iNos: inducible nitric oxide synthase; NSCs: neural stem cells; Pvh: paraventricular nucleus of the hypothalamus; ROS: reactive oxygen species; TNBS: trinitrobenzene sulfonic acid, Tlr: toll-like receptor; Tnf: tumor necrosis factor; Tregs: regulatory T cells; w: weeks; ↑: increased; ↓: decreased; ↔: no change.

In IBD patients, circulating chemokines and cytokines are significantly elevated, but no distinct cytokine profile distinguishes CD from UC [72]. In the sera of both UC and CD patients, levels of IL-1β, TNF, IL-1ra, IL-10, IL-6, C-X-C Motif Chemokine Ligand 1 (CXCL1, also known as growth related oncogene, GRO), and IL-8 are significantly elevated. Besides, UC patients exhibit increased levels of IL-17 and C-C Motif Chemokine Ligand 11 (CCL11, also known as eotaxin-1), whereas serum IFN-γ is elevated in CD [72]. In pediatric IBD patients, serum levels of cytokines and chemokines (IL-1β, IL-4, IL-6, IL-13, TNF, IL-1ra, IL-5, IL-7, IL-12, IL-8, CCL11, and CCL4) are increased and IL-7 was identified as a candidate biomarker for distinguishing CD from UC [73]. Noteworthy, an inflammatory cytokine signature can be detected years prior to diagnosis of UC, indicative for a systemic inflammatory pattern even at a presymptomatic disease stage [45]. As circulating mediators and inflammatory cells also contribute to IBD pathogenesis, this raises the question whether a systemic immune activation might be a primary cause, rather than a consequence, of chronic gut inflammation in IBD.

Overall, there is strong evidence for dissemination of local gut inflammation triggering systemic inflammatory responses, which facilitates CNS affection in IBD.

## 6. Routes from Peripheral Inflammation to the CNS in IBD

**Figure 1:**
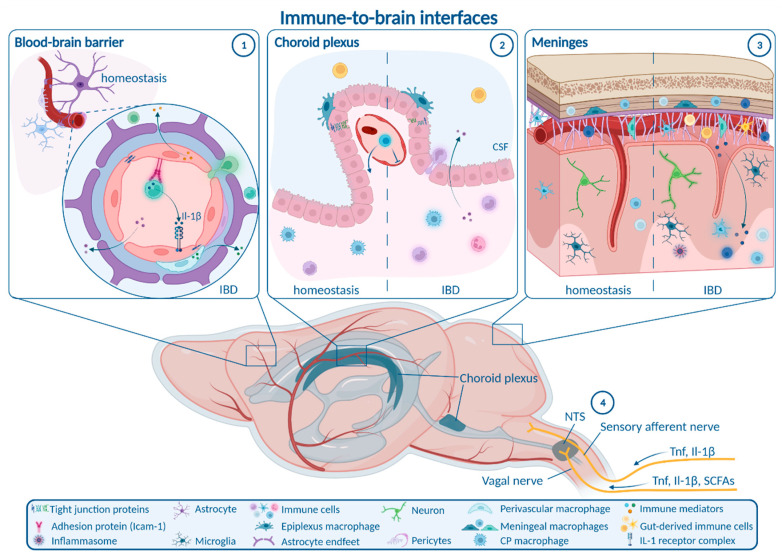
Alterations at immune-to-brain interfaces during IBD. (**1**) At the blood–brain barrier (BBB), downregulation of tight junction proteins and endothelial permeability mediate influx of inflammatory molecules and immune cells. Additional non-disruptive BBB changes comprise endothelial upregulation of adhesion proteins for the interaction and transmigration of circulating immune cells, as well as the release of mediators by endothelial cells and perivascular macrophages that modulate microglial and neuronal function. (**2**) At the choroid plexus (CP), decreased expression of tight junction proteins and increased fenestration followed by transient closure of the vascular barrier are observed, whereas permeability of the epithelial barrier is transiently enhanced. Numbers of stromal monocytes and neutrophils are increased during intestinal inflammation. (**3**) In the meninges, infiltration of gut-derived immune cells as well as activation of meningeal immune cells and the NLRP3 inflammasome can be detected. (**4**) Neural communication pathways linking gut inflammation and the brain include the afferent input via the vagal nerve to the nucleus tractus solitarii (NTS), and information from sensory afferent neurons stimulated in the periphery that provoke neural activation in different brain regions. Gut-derived immune cells (2) can enter the CSF via the CP or the meninges. CP: choroid plexus; CSF: cerebrospinal fluid; SCFA: short chain fatty acids; Tnf: tumor necrosis factor; Il-1β: interleukin-1β; NTS: nucleus tractus solitarii. Figure created with BioRender.com (accessed on 15 September 2022).

Chronic gut-derived systemic inflammation is able to take different anatomical routes connecting the gastrointestinal tract and the bloodstream with the CNS in order to provoke an inflammatory response within the brain (Figure 1).

### 6.1. Enteric, Autonomic and Sensory Nervous System Signaling

The peripheral (i.e., enteric, autonomic) and central nervous systems are tightly connected to form a bidirectional neural communication highway between the gastrointestinal tract and the brain. This route can be employed by inflammatory signaling towards the CNS directly from the gut, without requiring transition into the bloodstream [74]. A major transit route of gut-derived signals is the vagal nerve. Under homeostasis, vagal nerve afferents in the gut monitor key physiological parameters [75] and microbial metabolites like the SCFA butyrate, a surrogate for nutritional state [76]. These afferents project to the nucleus tractus solitarii (NTS) in the brainstem, directly activating neurons in the efferent dorsal motor nucleus of the vagal nerve to signal back to the gut, but also project to many other brain regions [77]. Under inflammatory conditions, vagal sensory neurons are able to sense Tnf and Il-1β, and signal inflammatory cues to the NTS by cytokine-specific electrophysiological patterns [78]. Moreover, early studies implicated the vagal nerve in CNS transmission of peripherally derived Il-1β in fever induction that was abrogated in rats by vagotomy [79]. The role of the vagal nerve in transmitting chronic gut inflammation in IBD towards the brain is not well elucidated and requires further investigation. In chronic mild gut inflammation caused by infection of mice with the parasite *Trichuris muris* (*T. muris*), anxiety-like behavior was reduced by systemic anti-inflammatory treatment, but not altered by vagotomy [80]. Besides a potential role of the vagal nerve in signaling of inflammatory cues to the brain, cholinergic efferent vagal nerve fibers can ameliorate gastrointestinal inflammation in TNBS-induced colitis [81]. Moreover, vagal nerve stimulation was reported to be beneficial both in depression and IBD, again pointing to the 10th cranial nerve as a major regulator of the gut–immune–brain axis [74,82].

A second neural route potentially involved in immune communication from the gut to the brain is the nociceptive system. IBD is associated with severe abdominal pain. In vitro data suggested that TNF in the supernatant of colonic biopsies from UC patients activated nociceptors on dorsal root ganglia neurons [83]. In line with this, IBD patients receiving TNF antagonists show a rapid and profound reduction in blood oxygen level dependent (BOLD) brain activity in functional magnetic resonance imaging (fMRI) after application of painful stimuli [84]. Strikingly, this CNS-mediated treatment response was present prior to the resolution of gastrointestinal inflammation [84]. These data suggest TNF-mediated upward signaling of gastrointestinal inflammation via the nociceptive afferent system contributes to functional CNS alterations in IBD. Therefore, neural communication pathways like the vagal nerve and the nociceptive system transmit gut inflammation to the brain.

### 6.2. Blood–Brain Barrier

The blood–brain barrier (BBB) controls the passage of circulating molecules and cells from the blood into the brain parenchyma. It consists of vascular endothelial cells connected by tight junctions and situated on a vascular basement membrane. The brain parenchyma is bordered by a layer of astrocytic end feet and their corresponding basement membrane. The perivascular space is located between the two basement membranes and contains perivascular macrophages and pericytes [85,86]. In most brain regions, the BBB prevents passive diffusion of hydrophilic molecules larger than 500 Da into the brain [52]. Thus, the BBB is by far less permeable than the GVB. However, several small regions located around the third and fourth ventricle, the so-called circumventricular organs (CVOs), exhibit more permeable endothelia to allow bidirectional interaction between the brain and the periphery, e.g., for endocrine signaling.

Systemic inflammation can modulate BBB function by different mechanisms [87]. Disruption of BBB tight junctions facilitates paracellular influx of inflammatory molecules and immune cells. In animal models of IBD, BBB disruption was investigated based on expression of tight junction markers and dye permeability assays, leading to heterogenous results. Reduction in the tight junction markers occludin and claudin-5 in the brains of mice with acute DSS-induced colitis was reported in one study [24], while this reduction required additional hypoxia in a second study [54]. Similarly, the permeability of Evans Blue-binding albumin (molecular weight 69 kDa) into the brain parenchyma of mice with acute DSS-induced colitis was increased [59] or unchanged [24], respectively. A recent time course analysis in mice treated with DSS for 3 days followed by 2 days of drinking water revealed a reduction in vascular zonula occludens-1 (Zo-1) after 3 days and a normalization after 5 days [53]. Strikingly, these mice showed an initial increase followed by a significant decrease in permeability for systemically administered fluorescent dextran (molecular weight 70 kDa), which also normalized after 5 days [53], indicating rapid dynamics of BBB regulation including a transient closure. Transient alterations of BBB permeability were also observed in TNBS-induced colitis showing enhanced permeation of Evans Blue-albumin after 1 day and a normalization at later time points [88]. Moreover, TNBS-induced colitis caused higher leakage of fluorescein (molecular weight 376 Da) into the brain parenchyma and different circumventricular organs [89,90]. Overall, these data indicate that disruptive changes in the BBB during the course of colitis may be of transient and highly dynamic nature. While changes in BBB permeability during the phase of acute colitis were demonstrated, data on chronic preclinical IBD are largely lacking.

Besides disruption and increased permeability of the BBB, systemic inflammation is able to induce so-called non-disruptive BBB alterations. This includes the upregulation of cellular adhesion molecules like intercellular adhesion molecule-1 (Icam-1) and vascular adhesion molecule-1 (Vcam-1) on BBB endothelia, facilitating the evasion of immune cells via the intact BBB [87]. Such mechanisms were barely addressed in experimental colitis models, except for one study reporting increased Vcam-1 mRNA in the brains of DSS-treated mice [61]. Infiltration of blood-derived immune cells contributes to parenchymal CNS inflammation due to either disruptive or non-disruptive BBB changes in distinct experimental models of IBD. Increased numbers of blood-derived monocytes and macrophages were observed in acute DSS-induced colitis [54,56,61], and to an even larger extent in chronic DSS-induced colitis [61]. Brain infiltration of neutrophils in acute DSS-induced colitis was reported either increased [56,61] or reduced [54]. In T cell transfer colitis, brain infiltration of monocytes and T cells was proposed [61]. However, many of these data must be interpreted with caution, as they are solely based on flow cytometry experiments and might be confounded by cells residing in the vascular lumen, the perivascular space, the meninges or the choroid plexus (CP). Future detailed structural studies must decipher the precise path of peripheral-derived immune cell localization and entry route.

Finally, systemic inflammation may result in direct inflammatory activation of BBB endothelial cells, which are able to secrete inflammatory mediators to modulate microglial and neuronal function [91]. During aging, upregulation of Vcam-1 on BBB endothelia inhibits neural progenitor cell (NPC) activity, mediates microglial activation, and induces an age-related impairment in hippocampal-dependent learning and memory [92]. These alterations were reversed by anti-Vcam1 antibody treatment [92]. The role of endothelia, rather as active neuroimmune players than a passive bystander, has not been addressed in the context of mucosal inflammation or experimental colitis.

Collectively, the permeability of the BBB during acute colitis is dynamically regulated, while insights on the BBB in chronic colitis as well as non-disruptive changes and immune activation of endothelial cells and perivascular macrophages during IBD need to be further analyzed in future studies.

### 6.3. Choroid Plexus and Blood–CSF Barrier

Besides alterations in BBB integrity, the CP has been identified as a gateway for pathogens, cells, and molecule transport into the CNS via the cerebrospinal fluid (CSF) [53,93,94]. The CP contains blood vessels with fenestrated endothelia as well as a layer of epithelial cells connected by tight junctions separating the CP from the CSF. Together, CP endothelia and epithelia form the blood–cerebrospinal fluid barrier (BCSFB). In addition, the CP contains a heterogeneous pool of immune cells, including T cells, B cells, dendritic cells, natural killer cells, lymphocytes, and two types of resident macrophages, one of which resembles brain parenchymal microglia [95].

In recent years, several findings shed light on the CP and the BCSFB as an important immune interface between the systemic circulation and the CNS. During aging and systemic inflammation, type I-interferon signaling in the CP becomes upregulated, which mediates glial cell activation and cognitive impairment [91,96]. Moreover, lipopolysaccharide (LPS)-induced systemic inflammation has the potential to trigger the release of extracellular vesicles by the CP, which propagate inflammation towards the brain [94].

Recently, the BCSFB has been thoroughly characterized in acute colitis induced by application of DSS for 3 days [53]. Strikingly, increased permeation of the CP vasculature after 1 day was followed by a transient closure of the vascular CP barrier after 3 days, whereas the tight junction marker Zo-1 was reduced between CP epithelial cells [53]. Together, these changes lead to a decrease in fluorescent tracer permeation into the CSF. Transient closure of the CP vascular barrier was orchestrated by the Wnt-β-catenin-pathway in endothelial cells and contributed to anxiety-like behavior and memory impairment [53]. These findings challenge the concept of general vascular barrier disruption by systemic inflammation, and suggest a contribution of the open CP vascular barrier to cognitive processing. Apart from the BCSFB, the role of the diverse CP immune cells during IBD and related CNS comorbidity is not well understood. The closure of CP vasculature in DSS-treated mice was accompanied by reduced numbers of CP macrophages and a rapid reduction in neutrophil infiltration [53]. Overall, acute DSS-induced colitis led to increased numbers of CD45+ leukocytes in the CP, but a further characterization of cell types was not performed [68]. Future studies focusing on the contribution of CP immune cell subsets and the BCSFB, especially in chronic experimental colitis models, are necessary.

In summary, the CP and the BCSFB may display a promising target for the treatment of IBD-associated neuropsychiatric comorbidity. This is further suggested by recent imaging data drawing a possible link between CP volume, BBB and BCSFB closure, and neuroinflammation in patients with depression [93].

### 6.4. Meninges

The meninges, covering the CNS surface, are divided into the leptomeninges (pia mater and arachnoidea mater) and the dura mater, both of which contain resident macrophage populations as well as diverse other immune cells under homeostasis [95]. A growing body of evidence underpins the relevance for meningeal immune signaling for CNS homeostasis. T cells in the meninges regulate neural activity and social behavior through IFN-γ that directly activates γ-aminobutyric-acid (GABA)-ergic neurons [97]. Additionally, meningeal γδT cells were recently shown to signal to glutamatergic neurons via Il-17a inducing anxiety-like behavior [98]. Interestingly, immunological interaction between the gut and the meninges was observed in stroke, where gut-derived CD11c+ myeloid cells were found to migrate to the meninges and CNS [99]. Moreover, T cells expressing gut-homing receptors have been shown to circulate in the CSF of patients with non-inflammatory neurological disease [100]. These cells might enter the CSF via the meninges or the CP.

In mice with chronic DSS-induced colitis, activation of the NACHT, LRR, and pyrin domain containing protein 3 (NLRP3) inflammasome in the meninges was proposed and linked to an increased infiltration of gut-derived T cells [64]. These findings indicate that the meninges may act as a gut-to-brain immune interface in IBD. 

Overall, distinct anatomical interaction pathways may contribute to the propagation of gut-derived systemic inflammation towards the CNS, subsequently triggering neuroinflammation.

## 7. Neuroinflammation in IBD Mouse Models

Once gut-derived peripheral inflammation propagates into the CNS, resident immune cells like astrocytes and microglia, the brain-resident macrophages, may acquire an inflammatory state and secrete cytokines and chemokines. Moreover, blood-derived immune cells and inflammatory factors are attracted to the brain. These changes are referred to as neuroinflammation. Neuroinflammatory changes were investigated in many studies on experimental models for IBD, leading to divergent results (Table 1).

### 7.1. Molecular Neuroinflammation in IBD Mouse Models

On a molecular level, elevated CNS concentrations of inflammatory mediators like Tnf, Il-1β, and Il-6 were detected in many studies (Table 1). A source of Il-1β is the NLRP3 inflammasome, an intracellular signaling complex triggering the activation of caspase-1, which cleaves pro-Il-1ß and pro-Il-18 into their active forms [101]. Activation of the NLRP3 inflammasome was observed in aged mice with chronic DSS-induced colitis [64]. Further inflammatory pathways potentially involved in IBD-linked neuroinflammation include activation of Toll-like receptor (Tlr) 2 and 4, which sense pathogen-associated molecular patterns (PAMPs), leading to nuclear factor ‘kappa-light-chain-enhancer’ of activated B-cells (NF-κB)-mediated inflammatory gene expression. Both in acute DSS- and DNBS-induced colitis, *Tlr2* and *Tlr4* expression was increased in the hippocampus [62,69]. Furthermore, the damage-associated molecular patterns (DAMPs) S100A9 and high-mobility group box 1 (HMGB1), as well as reactive nitrogen species (RNS), were suggested as potential therapeutic targets for neuroinflammation in IBD [56,59,70]. In summary, there is broad evidence for elevated levels of inflammatory mediators in the brain during gut-derived peripheral inflammation. However, their cellular source and involved inflammatory mechanisms are barely understood.

### 7.2. Cellular Neuroinflammation in IBD Mouse Models

On a cellular level, the activation of microglia as a potential driver of IBD-linked neuroinflammation was frequently proposed. Contrarily, few studies did not detect signs of microglial activation [61,63] challenging their role in IBD-associated CNS immune response. In these cases, microglia might be in a primed state and require a second hit to become activated. Potential second hits reported in amplifying neuroinflammation in experimental colitis models included irradiation [102], hypoxia [54], and water avoidance stress [103]. Noteworthy, findings on microglia in IBD should be interpreted with caution, as many studies were restricted to the analysis of immunoreactivity for the myeloid cell marker ionized calcium-binding adapter molecule 1 (Iba1) or Iba1+ cell number (Table 1), which do not allow clear distinction of parenchymal microglia from other brain macrophages and blood-derived peripheral myeloid cell subsets. Though few studies applied additional flow cytometry analyses to identify microglia or performed morphometric analyses to infer their activation, an in-depth characterization of microglial response in IBD is still lacking. Future studies will help to dissect the myeloid cell response in the CNS by next-genome sequencing and fate-mapping approaches.

The implication of astrocytes in neuroinflammation has attracted growing attention in recent years. In particular, a potentially neurotoxic reactive subpopulation of astrocytes termed A1 astrocytes was identified [104]. However, astrocytes are less well studied in IBD. In chronic DSS-induced colitis, expression of the pan-reactive astrocyte marker glial fibrillary acidic protein (Gfap) and the A1 astrocyte marker complement factor 3 (C3) was increased in the hippocampus and cortex [64]. Moreover, astrocytes exhibited morphological alterations (process extension and hypertrophy) suggestive for a reactive state [59]. The precise astrocytic response in IBD and the communication between astrocytes and microglia require further elucidation.

Besides brain-resident cells, infiltrating blood-derived immune cells are able to contribute to neuroinflammation. Their infiltration depends on spatiotemporal modulation of the BBB and is regulated in a highly dynamic fashion [53]. Peripheral inflammatory monocytes are attracted to the CNS by inflammatory cytokines and chemokines including Ifn-γ [105] and CC-chemokine ligand (Ccl) 2 [106]. A recent study proposed neutrophils as the main source of cerebral Tnf, and thus as a major driver of neuroinflammation during DSS-induced colitis [61]. Nevertheless, more thorough analyses regarding the regulation and spatiotemporal dynamics of blood-derived cell infiltration and the function and fate of blood-derived cells during IBD-related neuroinflammation are necessary to clarify their contribution to the CNS-associated comorbidity.

To summarize, different cell types are involved in IBD-associated neuroinflammation, but their precise roles and potential interactions are not yet sufficiently understood.

### 7.3. Spatiotemporal Regulation of Neuroinflammation in IBD Mouse Models

Several findings revealed a temporal variation in neuroinflammation in IBD. Inflammatory response correlated with the duration of acute DSS exposure and was less pronounced in relapsing-remitting chronic DSS paradigms [60], suggesting a partial immune tolerance or resilience induced in the CNS during chronic peripheral inflammation. In contrast, the infiltration of blood-derived monocytes and neutrophils was higher in chronic than in acute DSS-induced colitis [61]. A temporal decrease in inflammatory response was observed during recovery phases with normal drinking water after acute DSS [68] or TNBS application [71]. Nevertheless, acute colitis in adolescent mice caused long-lasting neuroinflammatory alterations even after a recovery phase of 5–7 weeks [62]. Collectively, these findings imply that neuroinflammation is dynamically regulated during gut-derived peripheral inflammation. Future studies will need to determine factors regulating the onset, duration and recovery of neuroinflammation during IBD and the influence of age on gut-derived immune responses in the CNS.

Besides temporal aspects of neuroinflammation in experimental colitis, a regionally specific CNS response must be taken into account. We previously reported a distinct regional immune fingerprint in mice with chronic peripheral inflammation caused by constitutive overexpression of human Tnf under the murine Tnf promoter [107]. Such an extensive regional characterization of neuroinflammation has not been performed in preclinical IBD models. Most studies focused on the cortex and the hippocampus as main regions of interest in depression and memory (Table 1). Only a few studies further addressed the hypothalamus due to its implication in coordination of the autonomous nervous and endocrine system and the amygdala due to its pivotal role in anxiety [63,68,108]. Interestingly, CVOs were differentially susceptible to acute DSS-induced colitis, with neuroinflammatory changes observed only in the subfornical organ and the median eminence, while other CVOs remained resilient [68]. Future studies will help to more thoroughly reveal microenvironmental susceptibility to gut-derived peripheral inflammation and identify mechanisms, by which more resilient brain regions are protected from developing neuroinflammation.

In summary, a growing body of evidence indicates neuroinflammation in chemically induced preclinical colitis models. However, data on IBD patients and its genetic mouse models are lacking. Moreover, the interaction of different cellular and molecular drivers of neuroinflammation needs to be precisely elucidated.

## 8. How Neuroinflammation Is Linked to Depression and Anxiety

CNS immune activation is a well-known phenomenon in depression and anxiety. Recent findings indicate that immune processes represent a key pathogenic driver rather than a pure epi-phenomenon in both conditions. This is supported by the notion that brain-resident immune cells including microglia and brain-resident CD4+ T cells are essential for neuronal homeostasis and physiological behavior [109,110,111]. Moreover, microglia were reported to mediate behavioral deficits in models of depression and anxiety induced by chronic unpredictable stress or early-life inflammation induced by intraperitoneal LPS injection [112,113,114]. Involved inflammatory pathways being potential therapeutic targets include microglia–astrocyte crosstalk via glutaminase-1 [115], the NLRP3 inflammasome [116,117] and the clearance of reactive oxygen species (ROS) via silent information regulator 2 homolog 1 (Sirt1)—nuclear factor erythroid 2-related factor 2 (Nrf2)—hemoxygenase 1 (Ho-1)—signaling [118,119]. 

Interestingly, modulation of the BBB is also implicated in the etiology of stress-induced depression and anxiety in chronic social defeat stress. In particular, stress-susceptibility and depression-like behaviors were dependent on downregulation of the tight junction marker claudin-5 (Cldn5) in the hippocampus and nucleus accumbens, which was orchestrated via Tnf and histone deacetylase 1 (Hdac1) and facilitated vascular influx of Il-6 into the brain parenchyma [120,121]. As chronic gastrointestinal inflammation was reported to cause BBB tight junction downregulation comparable to social stress, it might also represent a trigger for BBB-mediated depressive-like behavior. Furthermore, non-disruptive BBB alterations may contribute to anxiety-like behavior. Social stress-induced anxiety was mediated by Il1-receptor 1 producing endothelial cells, which were activated by Il-1β-expressing monocytes attracted to the BBB by microglia [122]. These findings suggest that endothelia closely interact with myeloid cells and may act as a gatekeeper to further develop neuropsychiatric symptoms. A better understanding of non-disruptive BBB changes in IBD is necessary to explore if such mechanisms are present during gastrointestinal inflammation. 

**Figure 2:**
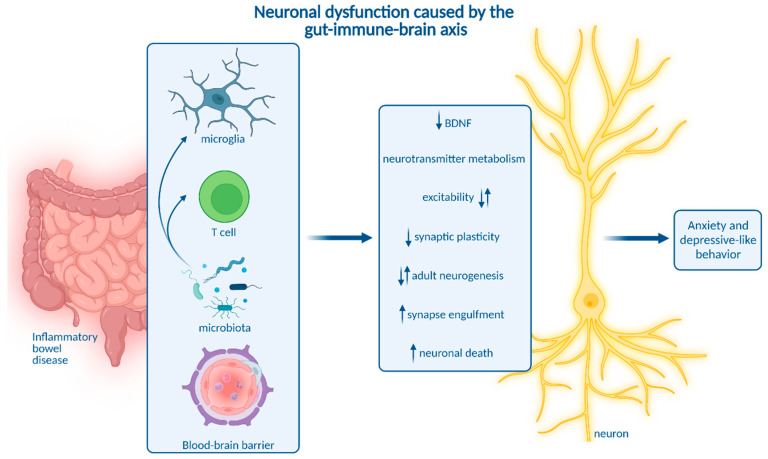
Impaired neuronal functions in inflammatory gut-to-brain communication. Microglia, peripheral immune cells and endothelia contribute to immune-mediated impairment of neuronal functions in IBD by different mechanisms. First, reduced neurotrophic signaling by BDNF is induced by neuroinflammation and observed in IBD. Second, inflammation interferes with neurotransmitter metabolism, e.g., resulting in reduced availability of serotonin. Third, electrophysiological properties of distinct neuronal populations are modified, and synaptic plasticity is reduced. Fourth, adult hippocampal neurogenesis is impaired. Fifth, increased microglial engulfment of synapses may lead to aberrant synaptic degradation. Finally, inflammatory signaling could induce neuronal cell death. Intestinal microbiota essentially contribute to neuronal alterations in IBD, either by augmenting inflammatory activation of immune cells or by direct influence on neurons. Disturbed neuronal function is the pathological correlate of behavioral changes and neuropsychiatric comorbidity in IBD. BDNF: brain derived neurotrophic factor; ↑: increased; ↓: decreased. Figure created with BioRender.com (accessed on 18 August 2022).

Though these and other studies strongly imply the relevance of neuroinflammation in depression and anxiety, they do not precisely delineate how neuroinflammation alter the function of neuronal circuits involved in behavioral and emotional regulation to ultimately trigger psychiatric symptoms. In the context of IBD, this might be mediated by several pathways (Figure 2).

First, CNS immune activation may compromise neuronal activity and synaptic transmission in key regions involved in anxiety and depression. Microglial activation was recently shown to reduce neuronal excitability in the dorsal striatum in a prostaglandin-dependent manner. Thus, targeting cyclooxygenase-1 (Cox-1)-mediated prostaglandin synthesis in microglia may alleviate depressive symptoms [112]. In the basolateral amygdala, inflammation induced by peripheral LPS application leads to increased glutamate release and projection neuron excitability, which was linked to depressive and anxiety-like behavior [123]. In line with these findings, TNBS-induced colitis reduced synaptic plasticity and elicited enhanced synaptic transmission in hippocampal glutamatergic neurons [71]. Interestingly, manganese-enhanced MRI indicated reduced hippocampal activity in chronic DSS-induced colitis [59]. Recently, inflammation-induced dysregulation of neuronal circuits was proposed to diminish inhibitory input from the prefrontal cortex and hippocampus on hypothalamic corticotropin releasing hormone (CRH) secretion. This, in turn, might aggravate both colitis and depressive-like behavior [48]. Together, electrophysiological properties and synaptic transmission of defined neuronal subtypes may be impaired during IBD-related neuroinflammation.

Additionally, CNS immune activation can shift neurotransmitter metabolism, in particular resulting in reduced availability of serotonin. Impaired serotoninergic signaling is involved in depression and is a major target for antidepressant treatment. In IBD, peripheral serotonin may act as a double-edged sword by augmenting mucosal inflammation [124], but protecting the enteric nervous system [125]. Of note, antidepressant serotonergic treatment positively influenced the disease course among patients with CD and UC, decreasing systemic pro-inflammatory cytokine levels [126]. Activated microglia express indoleamine 2,3-dioxygenase (IDO) to catabolize tryptophan into kynurenine instead of serotonin. Kynurenine is further processed into excitotoxic metabolites [127]. In acute DSS-induced colitis, IDO expression was increased in the prefrontal cortex [63]. Moreover, chronic colitis induced by infection with *T. muris* was accompanied by increased serum kynurenine levels [80]. These data suggest impaired tryptophan-serotonin metabolism in IBD, but a direct link between IBD-related neuroinflammation, impaired serotonergic signaling, and behavioral deficits has not yet been drawn.

Additionally, microglia can engulf and prune synapses. Microglial synaptic pruning is essential for proper brain development, but is aberrantly upregulated during neurodegeneration [128]. Intriguingly, the complement system, which is essentially involved in synaptic pruning, was recently implicated in stress-induced depressive-like behavior [129]. Moreover, microglia-synapse interactions were altered in models of depression in a spatiotemporally distinct manner. Early-life LPS-induced inflammation enhanced microglial engulfment of glutamatergic neuronal spines in the anterior cingulate cortex and thereby elicited depressive-like behavior in adolescence [113]. Depression and anxiety provoked by chronic unpredictable stress were linked to upregulation of microglial phagocytosis by neuronal colony-stimulating factor (CSF) 1, leading to reduced dendritic spine density on pyramidal neurons in the medial prefrontal cortex [114]. In contrast, early-life stress disturbed microglial engulfment of excitatory synapses in stress-sensitive CRH-expressing neurons in the paraventricular nucleus (PVN) of the hypothalamus [130]. The resulting activation of the hypothalamo–pituitary–adrenal axis impaired behavioral stress response. In IBD, synaptic clearance and involved pathways like the complement system are yet to be investigated. First insights indicate loss of Map2-positive dendritic nerve fibers in the cortex and hippocampus during chronic DSS-induced colitis in aged mice [64]. 

Besides structural dynamics of synapses, neuroinflammation in IBD might cause neuronal cell death. In line with this, the number of total neurons in the cortex and hippocampus of aged mice with chronic DSS-induced colitis was reduced [64]. Correspondingly, elevated expression of caspase 3 indicated increased apoptotic cell death in the brain during acute colitis, although this was not yet assigned to particular cell types [24]. Besides apoptosis, other kinds of cell death were not addressed in the CNS during IBD. Altogether, neuroinflammation in IBD could contribute to psychiatric symptoms by inducing structural alterations or degradation of synapses or cell death of neurons.

Impairing neuronal plasticity is another pivotal mechanism by which neuroinflammation might be able to mediate neuropsychiatric symptoms. Adult neurogenesis, the generation of new neurons in the brain throughout adult life, only occurs in few niches including the subgranular zone of the hippocampal dentate gyrus. Adult hippocampal neurogenesis is involved in learning, memory, and pattern separation [131]. However, impaired adult hippocampal neurogenesis is also linked to depression [132,133]. Importantly, there is broad evidence for impaired adult hippocampal neurogenesis during neuroinflammation. While homeostatic microglia maintain adult hippocampal neurogenesis [134], inflammatory cytokines like Tnf [135], as well as Il-1β [136] and peripheral inflammation induced by LPS administration [137], inhibit NPC proliferation and maturation. Intriguingly, elevated blood levels of Ccl11, a chemokine also observed in the serum of CD and UC patients [72,73], decreased adult hippocampal neurogenesis during aging-related peripheral inflammation [138]. In the context of IBD, a direct mechanistic link between neuroinflammation and adult hippocampal neurogenesis was not yet shown, but several studies in acute and chronic DSS-colitis show impaired adult neurogenesis in the hippocampus [60,62,65,67,139]. Noteworthy, acute and chronic DSS-induced colitis altered distinct aspects of adult hippocampal neurogenesis. Acute colitis increased progenitor cell proliferation, but dysregulated cell cycle kinetics, while chronic colitis led to reduced migration and functional integration of newly generated neurons [60]. Collectively, adult hippocampal neurogenesis is vulnerable towards peripheral and cerebral inflammation and may contribute to IBD-linked neuropsychiatric symptoms. 

Homeostatic brain functions, including adult neurogenesis, are governed by several trophic factors. One pivotal factor is the brain derived neurotrophic factor (BDNF), which signals via its receptor tyrosine receptor kinase b (Trkb). BDNF supports the release of neurotransmitters as well as the expression and function of neurotransmitter receptors and ion channels [140]. Moreover, BDNF augments synaptic plasticity and adult neurogenesis [140]. Interestingly, reduced BDNF levels were linked to depression [116], and a major mode of action of antidepressant drugs was recently revealed to be the amplification of BDNF-Trkb-signaling [141]. Of note, there is evidence for reduced BDNF signaling during neuroinflammation. The expression of BDNF is suppressed by Il-1β [142]. Furthermore, astrocyte-derived Il-33 was reported to reduce BDNF levels in the amygdala, which was linked to impaired signaling of GABAergic neurons [143]. In line with this, BDNF levels in the brain were reduced in acute and chronic DSS-induced as well as in DNBS-induced colitis [56,65,67,69]. Treatment with liver hydrolysate rescued neuroinflammation and depressive-like behavior in acute DSS-induced colitis, putatively by inducing BDNF expression via adenosine monophosphate-activated protein kinase (AMPK) [67]. Though neuroinflammation and reduced BDNF levels were only coincident and not causally linked in IBD models, impaired BDNF signaling might be triggered by neuroinflammation and contribute to depression and anxiety in IBD.

In summary, neuroinflammation can trigger neuronal dysfunction via a plethora of distinct mechanisms, thereby mediating neuropsychiatric comorbidity in IBD. Though many of these potential mechanisms were described in animal models for IBD, a major limitation of most studies is the lack of a causal relation between coinciding neuroinflammation and depressive-like behavior. Only marginal data supporting this causality were generated in pharmacological studies. Inhibition of the DAMP S100a9 alleviated DSS-induced colitis, neuroinflammation, and behavioral impairment [56]. However, these effects might be explained solely by the reduction in colitis rather than interference with immune gut-to-brain communication. Interestingly, systemic inhibition of RNS did not affect TNBS-induced colitis but diminished hippocampal Tnf levels and reversed depressive-like behavior [70]. Moreover, local intracerebroventricular administration of the antibiotic and immune modulatory drug minocycline reduced microglial activation and normalized synaptic plasticity in TNBS-induced colitis [71]. Though these findings link neuroinflammation and neuronal dysfunction in the context of IBD, the applied treatment paradigms are unspecific. Thus, more specific approaches are required to investigate the causal link between individual immune cell types, neuronal dysfunction and neuropsychiatric symptoms in IBD.

## 9. Impact of Microbiota on Neuroinflammation and Neuropsychiatric Disease

Having highlighted different routes of transmission from chronic gastrointestinal inflammation into the systemic circulation and into the CNS as well as consecutive neuronal dysfunction and behavioral impairment, we will shed some light on the role of gut microbiota in the gut-immune-brain interplay. The intestinal microbiota is substantially involved in gastrointestinal inflammation and neuropsychiatric diseases [44,108,144,145]. Therefore, microbiota and their metabolites are emerging key players in the gut-immune brain axis during IBD. This notion has encouraged several probiotic treatment approaches. Indeed, application of probiotic bacterial strains alleviated DSS-induced colitis, reduced systemic and CNS cytokine levels, induced micro-RNA expression related to restoring inflammation-associated microbiota dysbiosis, and improved depressive and anxiety-like behavior [146,147,148,149]. However, it is unclear whether effects of probiotic treatments were solely indirect based on the reduction in gut inflammation or also directly interfered with inflammatory gut-to-brain communication or neurons. It is important to note, that changes in the gut microbiota may be cause or consequence of intestinal inflammation and modulate neuropsychiatric symptoms via affecting neuroinflammation, but also exert direct effects on neurons. We will therefore highlight different modes of action which affect the CNS during IBD-related dysbiosis.

First, microbiota and microbiota-derived molecules actively shape the CNS immune landscape, and the presence of a complex microbiota is essential for microglia activation and function [148,150,151,152]. Among other CNS-associated myeloid cell types, commensal microbiota strongly influence CP macrophages, while their impact on perivascular and meningeal macrophages is moderate [148]. Different microbial metabolites were described to modulate microglia. First, microbiota-derived SCFAs signaling via the free fatty acid receptor 2 (Ffar2) were implicated in microglial maturation and function [151]. A recent differential analysis of distinct SCFAs revealed acetate to be a key regulator of microglial metabolism and phagocytosis [151]. In the context of multiple sclerosis, the SCFA propionate was shown to induce regulatory Treg activation, whereas pro-inflammatory Th1 and Th17 responses were diminished [153]. Moreover, bacterial metabolites of dietary tryptophan signal via the aryl hydrocarbon receptor to reduce microglial inflammatory activation of astrocytes [154]. In the context of IBD, UC patients with comorbid depression or anxiety showed a distinct intestinal bacterial profile linked to reduced blood levels of the metabolites 2ʹ-deoxy-D-ribose and L-pipecolic acid [44]. Intriguingly, substitution of these metabolites in mice alleviated DSS-induced colitis as well as cytokine levels in the blood and brain, but also ameliorated anxiety and depressive-like behavior [44]. These findings suggest a role of microbial metabolites in gut-immune-brain communication during IBD. Future studies addressing the modulation of neuroinflammation and behavior in mouse models for experimental colitis by the above-mentioned metabolites will improve our understanding of microbial influence on IBD-related CNS morbidity. 

In addition to gut bacteria, mucosal fungi were shown to promote Th17 cell activation, which promotes social behavior by direct signaling to neurons via Il-17 [42]. Collectively, intestinal microbiota are able to modulate systemic and CNS immune responses in IBD and could thereby contribute to IBD-related CNS comorbidity.

Besides indirect immune-mediated effects of microbiota and derived metabolites, they have the potential to exert immune-independent effects on neurons via different pathways. Gut bacteria-derived outer membrane vesicles (OMVs) can cross the intestinal barrier and even the BBB, enabling a shuttled transfer of microbial bioactive molecules to the brain [155]. Interestingly, bacterial OMVs were differentially taken up by neurons with a regionally distinct affinity [155]. Though their influence on neuronal function is unknown, bacteria-derived OMV cargo uptake may be enhanced in IBD due to gut barrier and BBB dysfunction and may be a complementary part of the gut-to-brain communication.

Moreover, intestinal microbiota are involved in neurotransmitter metabolism. Expansion of *Bacteroides* species contributes to depressive-like behavior and impaired hippocampal neurogenesis by regulating tryptophan and neurotransmitter metabolism [145]. Interestingly, *Bacteroides* species were also linked to the development of colitis [156]. Apart from that, gut microbiota are a major source of the neurotransmitter serotonin. Reduced serotonin abundance as a potential pathogenic mechanism driving depression may be caused by microbial dysbiosis and impaired serotonin production in the gastrointestinal tract [157]. 

In addition, gut microbiota influence the expression of micro-RNA (miRNA) in the gut and in different brain regions, which is associated with depression and anxiety in mice [158,159]. Interestingly, differential miRNA expression associated with microbiota dysbiosis distinguishes IBD patients and healthy individuals. MiRNAs are thus suggested as biomarkers and promising targets to treat intestinal inflammation [160].

Compromised gut microbiota in IBD might therefore contribute to the pathophysiology of concomitant anxiety and depression via affecting neurotransmitter homeostasis and miRNA expression.

Besides prototypical neurotransmitters and miRNA, microbial metabolites have the potential to actively signal to neurons. The phenolic compounds phenyl sulfate, pyrocatechol sulfate, and 3-(3-sulfooxyphenyl)propanoic acid as well as indoxyl sulfate were implicated in synaptic remodeling and fear extinction learning [161]. Moreover, δ-valerobetaine produced by diverse bacterial species modulates inhibitory synaptic transmission and neuronal network activity independent of microglia, preventing age-related decline in cognitive function [162]. SCFAs are able to act locally in the ENS and enhance enteric neuronal survival and neurogenesis potentially acting via the 5-hydroxytryptamine type 4 (5-HT4) receptor [163]. SCFAs were also found in the brain, but their levels were not altered during chronic DSS-induced colitis [59].

In acute DSS-induced colitis, bacteria of the *Lachnospiraceae*, *Ruminococcaceae* and *Muribaculaceae* families were observed to be associated with anxiety and depressive-like behavior. Fecal microbial transfer of colitis-characteristic gut microbiota composition into germ free or antibiotic-treated mice was sufficient to transmit behavioral abnormalities without inducing neuroinflammation, suggesting that immune-independent mechanisms promote microbiota-mediated behavioral alterations in IBD [108]. 

Altogether, gut microbiota likely contribute to the development of neuroinflammation and neuropsychiatric symptoms in IBD. Future studies will need to identify essential bacterial strains and the mechanisms they employ to alter neuroinflammation or directly modify neuronal function specifically during chronic gastrointestinal inflammation.

## 10. Conclusions

In the present review, we propose a step-by-step pathogenetic course leading to neuropsychiatric comorbidity in IBD, starting with dissemination of gastrointestinal inflammation into the systemic circulation. Hence, neuroinflammation is triggered via distinct anatomical routes and results in neuronal dysfunction by a variety of mechanisms. Anxiety and depressive-like behavior predominantly represent the phenotypical correlate of neuronal dysfunction. Numerous steps linked to the gut–immune–brain axis were analyzed in preclinical models of IBD, yet their causal relation remains to be further consolidated in future studies. By now, many observations may be explained by reciprocal causation. For example, mucosal inflammation may result in neuronal dysfunction or damage via immune-independent mechanisms involving gut microbiota, and neuronal damage might result in inflammatory response within the CNS. 

The present literature largely focuses on the disruption of the blood–brain barrier as the entry route for colitis-induced systemic inflammation into the brain. In the present review, we comprehensively highlight the emerging dynamic and active role of the BBB in immune-to-brain communication, which also has to be addressed in the context of IBD. Furthermore, we focus on other emerging immune-to-brain interfaces including the meninges and the CP. As the CNS and its associated borders harbor a rich landscape of different immune cells, future studies will need to dissect immune cell diversity during IBD-related CNS pathology. More sophisticated approaches including cell-specific gene targeting of microglia, astrocytes or endothelial cells will reveal if, and which, cell types or specific inflammatory mechanisms contribute to impaired neuronal function and are causative for neuropsychiatric deficits in IBD. Moreover, the current state of knowledge about IBD-related CNS comorbidity is predominantly based on chemically induced colitis in rodents. Future studies on transgenic models for experimental colitis with further implication of important IBD-characteristic features, will extend our molecular understanding of inflammatory gut-to-brain communication. Importantly, a comparative analysis of different preclinical IBD models will reveal the heterogeneity of the gut–immune–brain axis dependent on particular inflammatory conditions, and also reflect the heterogeneity within the clinical spectrum of IBD patients. Though difficult to obtain, CSF or post-mortem brain tissue analyses of IBD patients are necessary to translate preclinical findings to humans. Overall, an in-depth characterization of the gut–immune–brain axis will aid to identify targets for a more effective treatment of depression, anxiety, but also other neurological comorbidities of IBD including Parkinson disease.

## Data Availability

Not applicable.

## Abbreviations

AMPK: adenosine monophosphate-activated protein kinase; APC: antigen presenting cell; ATP: adenosine triphosphate; BBB: blood brain barrier; BCSFB: blood–cerebrospinal fluid barrier; Bdnf: brain-derived neurotrophic factor; C3: complement factor 3; CCL: C-C Motif Chemokine Ligand; CD: Crohn’s disease; Cldn5: claudin-5; CNS: central nervous system; COX-1: cyclooxygenase-1; CRH: corticotrophin releasing hormone; CSF: cerebrospinal fluid; CP: choroid plexus; CVOs: circumventricular organs; Cx: Cortex; CXCL: C-X-C Motif Chemokine Ligand; d: day; DAMPs: damage-associated molecular pattern; Dcx: doublecortin; DNBS: dinitrobenzene sulfonic acid; Drn: dorsal raphe neucleus; DSS: dextran sulfate sodium; EGCs: enteric glial cells; ENS: enteric nervous system; Erc: entorhinal cortex; Ffar2: free fatty acid receptor 2; GABA: γ-aminobutyric acid; Gfap: glial fibrillary acidic protein; GRO: growth related oncogene; Gsdm: gasdermin; GSH: glutathione; GVB: gut vascular barrier; Hc: hippocampus; Hdac1: histone deacetylase 1; HIF-1α: hypoxia inducible factor-1α; HMGB1: high-mobility group box protein 1; Ho-1: hemoxygenase-1; Ht: Hypothalamus; Iba1: ionized calcium-binding adaptor molecule 1; IBD: inflammatory bowel disease; Icam-1: intracellular adhesion molecule-1; IDO: indoleamine 2,3-dioxygenase; IECs: intestinal epithelial cells; IFN: interferon; IL: interleukin; iNos: inducible nitric oxide synthase; i.r.: intrarectal; IR: immunoreactivity; LPS: lipopolysaccharide; Mϕ: macrophage; Map2: microtubuli-associated protein 2; Me: median eminence; MeA: medial amygdala; Men: meninges; n.a.: not available; NAc: Nucleus accumbens; NF-κB: nuclear factor ‘kappa-light-chain-enhancer’ of activated B-cells; NLRP3: NACHT, LRR, and pyrin domain containing protein 3; NMRI: Naval Medical Research Institute; NPC: neural progenitor cell; Nrf2: nuclear factor erythroid 2-related factor 2; NSCs: neural stem cells; NTS: nucleus tractus solitarii; OMV: outer membrane vesicle; OXA: oxazolone; PAMPs: pathogen-associated molecular patterns; PRRs: pattern recognition receptors; Pvh: paraventricular nucleus of the hypothalamus; PVN: paraventricular nucleus; RNS: reactive nitrogen species; ROS: reactive oxygen species; SCFAs: short chain fatty acids; Sirt1: silent information regulator 2 homolog 1; Th: T helper; TNBS: trinitrobenzene sulfonic acid; TLR: Toll-like receptor; TNF: tumor necrosis factor; Treg: regulatory T cell; Trkb: tyrosine receptor kinase b; UC: ulcerative colitis; Vcam-1: vascular adhesion molecule-1; w: weeks; ↑: increased; ↓: decreased; ↔: no change.
